# Probing ECM remodeling in idiopathic pulmonary fibrosis via second harmonic generation microscopy analysis of macro/supramolecular collagen structure

**DOI:** 10.1117/1.JBO.25.1.014505

**Published:** 2019-11-29

**Authors:** Darian S. James, Alexander N. Jambor, Hsin-Yu Chang, Zachary Alden, Karissa B. Tilbury, Nathan K. Sandbo, Paul J. Campagnola

**Affiliations:** a University of Wisconsin–Madison, Department of Biomedical Engineering, Madison, Wisconsin, United States; b University of Wisconsin–Madison, Division of Allergy, Pulmonary, and Critical Care Medicine, Madison, Wisconsin, United States

**Keywords:** second harmonic generation, collagen, fibrosis, scattering, polarization

## Abstract

Idiopathic pulmonary fibrosis (IPF) is a progressive disease with poor prognosis with short lifespan following diagnosis as patients have limited effective treatment options. A fundamental limitation is a lack of knowledge of the underlying collagen alterations in the disease, as this could lead to better diagnostics, prognostics, and measures of treatment efficacy. While the fibroses is the primary presentation of the disease, the collagen architecture has not been well studied beyond standard histology. Here, we used several metrics based on second harmonic generation (SHG) microscopy and optical scattering measurements to characterize the subresolution collagen assembly in human IPF and normal lung tissues. Using SHG directional analysis, we found that while collagen synthesis is increased in IPF, the resulting average fibril architecture is more disordered than in normal tissue. Wavelength-dependent optical scattering measurements lead to the same conclusion, and both optical approaches are consistent with ultrastructural analysis. SHG circular dichroism revealed significant differences in the net chirality between the fibrotic and normal collagen, where the former has a more randomized helical structure. Collectively, the measurements reveal significant changes in the collagen macro/supramolecular structure in the abnormal fibrotic collagen, and we suggest these alterations can serve as biomarkers for IPF diagnosis and progression.

## 1 Introduction

Idiopathic pulmonary fibrosis (IPF) is a form of interstitial lung disease that is a progressive disorder characterized by unrelenting scarring and stiffening of the lungs that leads to ∼34,000 deaths each year. The interstitial fibrosis is characterized by spatial and temporal heterogeneity; for example, areas of dense collagen accumulation (old scar) are juxtaposed with fibroblastic foci (new scar formation).^1^ As scarring increases, efficient lung function decreases, compromising the uptake of oxygen by blood, resulting in shortness of breath, limited exercise capability, and daily cough. The median survival for patients diagnosed with IPF is typically 3 to 5 years post-diagnosis.

Currently, IPF is clinically diagnosed using a combination of tests including chest x-ray, high-resolution computed tomography (HRCT), lung biopsy, and lung function tests. These techniques often lack the sensitivity and specificity needed to examine the extracellular matrix (ECM) changes and their role in IPF progression. For example, when the classic radiographic pattern is present, IPF can be diagnosed via HRCT.^2^ However, this pattern is not always uniquely defined due to the heterogeneity of the disease. Moreover, no radiographic method can probe the collagen morphology changes that are an integral part of the pathology. Biopsy followed by histology remains the gold-standard for IPF diagnosis; however, there is a significant risk of associated morbidity and longitudinal samples cannot be taken.^3^

There remains a clear need for better diagnostics as well as prognostic indicators. We suggest that probing the underlying macro/supramolecular changes in collagen presents such a new direction. Perhaps counterintuitively, the specific collagen changes in this fibrosis have not received significant attention beyond conventional H&E staining, showing increased deposition. As an alternative, second harmonic generation (SHG) microscopy offers considerable opportunities to study collagen alterations and provide insight into both disease etiology and progression. We previously showed that SHG combined with machine learning based on two-dimensional wavelet transforms/principal component analysis of the fiber morphology classified normal and IPF tissues with near 100% accuracy.^4^ In addition, we found that the collagen/elastin balance increased, consistent with increased clinical stiffness and difficulty breathing.

SHG microscopy can also be used to probe subresolution aspects of collagen architecture. For example, SHG polarization-resolved methods [e.g., linear SHG polarization analysis (P-SHG) and circular dichroism (SHG-CD)] can extract information on the helical structure of collagen (e.g., α-pitch angle and triple helical chirality).^5^ For example, we previously showed that collagen molecular aspects were different in human ovarian cancer from normal ovarian tissues.^6^ This is also relevant for IPF as there have been earlier reports using immunostaining that showed the Col I and Col III isoform balance changes during disease progression and is also different from that of normal tissue.^7^^–^^9^ Moreover, these isoforms have different pitch angles, and we have shown that these can be delineated by linear polarization analysis and application of the single-axis molecular model.^10^^,^^11^ Additionally, analysis of the SHG directional response, i.e., the forward-backward ratio (F/B), yields data on the subresolution size and packing. In this study, we used these SHG metrics along with bulk optical property measurements to characterize the macro/supramolecular structure of IPF and normal human lung tissues. Collectively, these optical readouts provide new insight into the collagen alterations in IPF that have been previously unattainable by other methods.

## 2 Materials and Methods

### 2.1 Human Tissues

All tissue samples (de-identified) were obtained from lung transplant recipients at UW Hospital Madison, Wisconsin, under a current IRB-approved protocol. The normal tissues were from pathologist-defined normal adjacent tissue from biopsies of patients without fibrotic lung disease. All IPF samples were from patients with advanced or highly progressed IPF. Tissues were fixed in 4% formalin and sectioned using a vibratome (Leica VT1200) to ∼200  μm thickness. We have shown that fixation does not significantly alter the fibril structure.^12^ After sectioning, the tissues were stored at 4°C in phosphate-buffered saline (PBS) for conventional SHG imaging or optically cleared by immersion in 50% glycerol overnight to reduce scattering-induced de-polarization effects for SHG polarization-resolved imaging. For imaging, samples were mounted on glass slides in PBS or glycerol with #1.5 coverslips and nail polish to seal the slides. A total of three normal and four IPF-independent patient samples were imaged and also used for optical property measurements.

### 2.2 Collagen Concentration Assay and α-SMA Staining

Using a Sirius Red Collagen Detection Kit (catalog no. 9062, Chondrex, Redmond, Washington), collagen concentration of collagen type I standards (8, 16, 31.5, 63, 125, 250, and 500  μg/ml solutions), blanks (acetic acid only), and our test samples (lung tissues) were extracted, in accordance with the manufacturer’s instructions. Each of the lung tissue samples was homogenized in 1  mg/mL pepsin in 0.05-M acetic acid and incubated for 10 days at 4°C. After collagen digestion and Sirius Red staining, the supernatant was collected, and the total collagen concentration was detected. A Tecan Infinite M1000 Plate Reader was used to measure the optical density (OD) at 530 nm to obtain absorbance readings from the standards, blanks, and lung samples. We subtracted the blank OD values from the standards and test samples. Then, we plotted the OD values of the standard curve using linear regression analysis and then calculated the collagen concentration (μg/ml) of the lung tissues. Three tissues per group, each run in duplicate, were analyzed.

α-smooth muscle actin (SMA) expression was imaged to identify fibrotic regions to be imaged by SHG. Samples were fixed in 4% formaldehyde, blocked in 1% BSA in PBS, permeabilized with 0.1% Triton X-100 for 30 min, and then incubated overnight at 4°C with the primary antibody, anti-α-SMA (monoclonal, mouse). Secondary staining was performed using goat anti-mouse IgG2a Alexa Fluor 594 (ThermoFisher, 1:1000 dilution) and then incubated overnight, then mounted in PBS and imaged by two-photon excited fluorescence (TPEF). The two-photon excitation laser wavelength was 780 nm, and the emission collection was centered at 632 nm with a bandpass filter.

### 2.3 SHG Microscope System

The details of the SHG microscope have been described elsewhere^13^^,^^14^ and are only briefly described here. The system consists of a laser scanning unit (FluoView 300; Olympus, Melville, New York) mounted on an upright microscope (BX61; Olympus, Tokyo, Japan), where the excitation source is a mode-locked titanium sapphire laser (Mira; Coherent, Santa Clara, California). Imaging was performed with a fundamental laser wavelength of 890 nm for SHG forward-backward (F/B) and P-SHG analysis and 780 nm for SHG-CD; the shorter wavelength for the latter provides greater sensitivity.^5^ Average powers at the focus were ∼30 to 50 mW using a 40× 0.8 NA water immersion lens (LUMPlanFL; Olympus, Tokyo, Japan) and a 0.9 NA condenser. The resulting lateral and axial resolutions were ∼0.7 and 2.5  μm, respectively. Forward- and backward-directed SHG emission was collected using matched photon-counting detectors (7421 GaAsP; Hamamatsu, Hamamatsu City, Japan), where the collection efficiencies were calibrated as before using fluorescent beads.^13^ The SHG wavelengths (445 and 390 nm) were isolated with the respective 10-nm-wide bandpass filters (Semrock, Rochester, New York). The excitation wavelength was confirmed using a fiber-optic spectrometer (Ocean Optics, Dunedin, Florida). Fields of view were 170×170  μm for SHG F/B and 85×85  μm for both SHG-CD and P-SHG and were acquired with scanning speeds of 2.71  s/frame with three-frame Kalman averaging. The power was controlled by an electro-optic modulator (ConOptics, Danbury, Connecticut) run by a custom LabVIEW program (National Instruments, Austin, Texas), interfaced with the FluoView scanning system using a data acquisition card (PCI-6024E; National Instruments).

Linear polarization was obtained using a half-wave plate to define the state entering the microscope, and the desired linear rotation at the focal plane was achieved using a liquid-crystal rotator (LCR; Meadowlark Optics, Frederick, Colorado) mounted in the infinity space.^14^ Circular polarization is achieved with a quarter-wave plate after the LCR, where left- and right-handed states are achieved with 90 deg of linear rotation by the LCR.^14^ The linear and circular polarization states were validated as previously described by imaging cylindrically symmetric giant vesicles.^5^^,^^14^ The polarization control was also run by a custom LabVIEW program interfaced to the FluoView scanning system.

### 2.4 SHG Polarization Analyses

#### 2.4.1 Helical pitch angle analysis

Polarization-dependent measurements were performed as previously described,^10^ where images were taken every 10 deg through 180 deg of rotation. Here, the method was applied to both optically cleared normal and IPF tissues, where clearing is required as scattering rapidly scrambles the excitation polarization.^15^ We had also showed that using thin tissues (∼10  μm) this process does not affect the polarization response. The α-helical pitch angle is extracted^10^ by combining the pixel-based generic model^16^ with the single-axis molecular model.^11^ As previously, we determined the pitch angle, $\theta_{p}$, through analysis of the symmetry reduced tensor elements:

$$\theta_{p}=\tan^{-1}\sqrt{2/b}=\tan^{-1}\sqrt{2/(\chi_{ZZZ}^{\left(2\right)}/\chi_{ZXX}^{\left(2\right)}).}$$ (1)

#### 2.4.2 Second harmonic generation-circular dichroism

SHG-CD analysis was used to interrogate the overall chirality of the human lung tissues; the method has been described previously.^5^ In brief, images of IPF and normal lung tissues were taken using left- and right-handed (LH and RH) circularly polarized (CP) laser excitation and were obtained 30-μm deep into the optically cleared tissues to avoid boundary effects. To account for variations in intensity in the different samples, we report the normalized SHG-CD response defined as

$$I_{\mathrm{SHG}-\mathrm{CD}}=\frac{\left|I_{(2\omega )\mathrm{LHCP}}-I_{(2\omega )\mathrm{RHCP}}\right|}{[I_{(2\omega )\mathrm{LHCP}}+I_{(2\omega )\mathrm{RHCP}}]/2},$$ (2)

where $I_{2(\omega )\mathrm{LHCP}}$ and $I_{2(\omega )\mathrm{RHCP}}$ represent the integrated pixel intensities of the SHG images of LHCP and RHCP, respectively. This is calculated on a pixel basis, where we first set a threshold mask above the noise background to identify nonzero pixel values. Absolute values were summed across the entire field of view as the sign of CD response will depend on fiber orientation.^5^^,^^17^^,^^18^

### 2.5 Bulk Property Measurements

The spectral dependence of the single scattering anisotropy, g, and the scattering coefficient, $\mu_{s}$, was determined using the setup previously reported by Hall et al.^19^ The scattering coefficient, $\mu_{s}$, is determined by on-axis attenuation measurements through the Beer–Lambert law:

$$I=\alpha I_{0}e^{-d(\mu_{s}+\mu_{a})},$$ (3)

where I is the transmission with the sample, $I_{0}$ is the transmission without the sample, α is the factor for losses due to refractive index mismatches, d is the tissue thickness, $\mu_{s}$ is the scattering coefficient, and $\mu_{a}$ is the absorption coefficient.^19^ Since lung is a collagen-rich tissue, $\mu_{a}\ll \mu_{s}$ and can be considered negligible.^20^

The scattering anisotropy, g, is first determined by goniometry, where the angular distribution of increasing scattering light is measured from 0 deg to 180 deg through the use of a rotating photodiode detector on a motorized stage and fit to the Henyey–Greenstein phase function (valid for most tissues):

$$p(\theta )=a\frac{1-g_{ef}^{2}}{{\left(1+g_{ef}^{2}-2g_{ef}\;\cos\;\theta \right)}^{\frac{3}{2}}}.$$ (4)

Multiple scattering broadens the angular response and yields a lower measured anisotropy, $g_{ef}$. We therefore developed a Monte Carlo simulation framework to extract the true anisotropy $g_{\text{single}}$ from the measured $g_{ef}$.^19^ $g_{\text{single}}$ is associated with the scattering directionality and structural organization of the tissue on a scale from 0 to 1, with the following limits: g=0, isotropic scattering, corresponding to randomly organized structure; and g=1, all forward-directed scattering, and associated with a highly organized structure relative to the excitation wavelength. The independent determination of g and $\mu_{s}$ also yields the reduced scattering coefficient, $\mu_{s}^{\prime }$ (which is used as an input parameter for the Monte Carlo simulations for SHG directionality):

$$\mu_{s}^{\prime }=\mu_{s}(1-g).$$ (5)

### 2.6 Determination of SHG Emission Directionality

The emission or creation directionality ($F_{\mathrm{SHG}}/B_{\mathrm{SHG}}$) is reflective of the fibril assembly, i.e., size and packing in the axial direction^21^ and is determined by measuring the depth-dependent measured F/B through image stacks of ∼100  μm of thickness. The depth-dependent response then results from a convolution of the $F_{\mathrm{SHG}}/B_{\mathrm{SHG}}$ with scattering ($\mu_{s}^{\prime }$) of the SHG signal at $\lambda_{\mathrm{SHG}}$. As previously described, a Monte Carlo simulation framework^22^ (modified for SHG from MCML)^23^ using the measured bulk optical properties (Sec. 2.5) extracts the emission directionality $F_{\mathrm{SHG}}/B_{\mathrm{SHG}}$.^20^^,^^22^ The SHG emission directionality was determined both across the whole field of view as well as locally to investigate the extent of heterogeneity in the tissues.^24^ Here, a 15×15  pixel patch area (corresponding to ∼5.3×5.3  μm) was used as it optimally captures the fiber and fiber bundle structures of the tissue and is adequately robust to Poisson noise. The number of stacks for this analysis was 34 and 75 for the IPF and normal tissues, respectively.

### 2.7 Statistical Analysis

One-way analysis of variance (ANOVA) with Student’s t-tests (polarization-resolved SHG, SHG-CD) and Tukey’s honest significant difference *post-hoc* tests (bulk optical property measurements, all other SHG methods) were performed in Origin 9.1 (OriginLab, Northampton, Massachusetts). p-values less than α=0.05 were considered statistically significant.

## 3 Results

### 3.1 Assessment of Overall Collagen Assembly

**Fig. 1:**
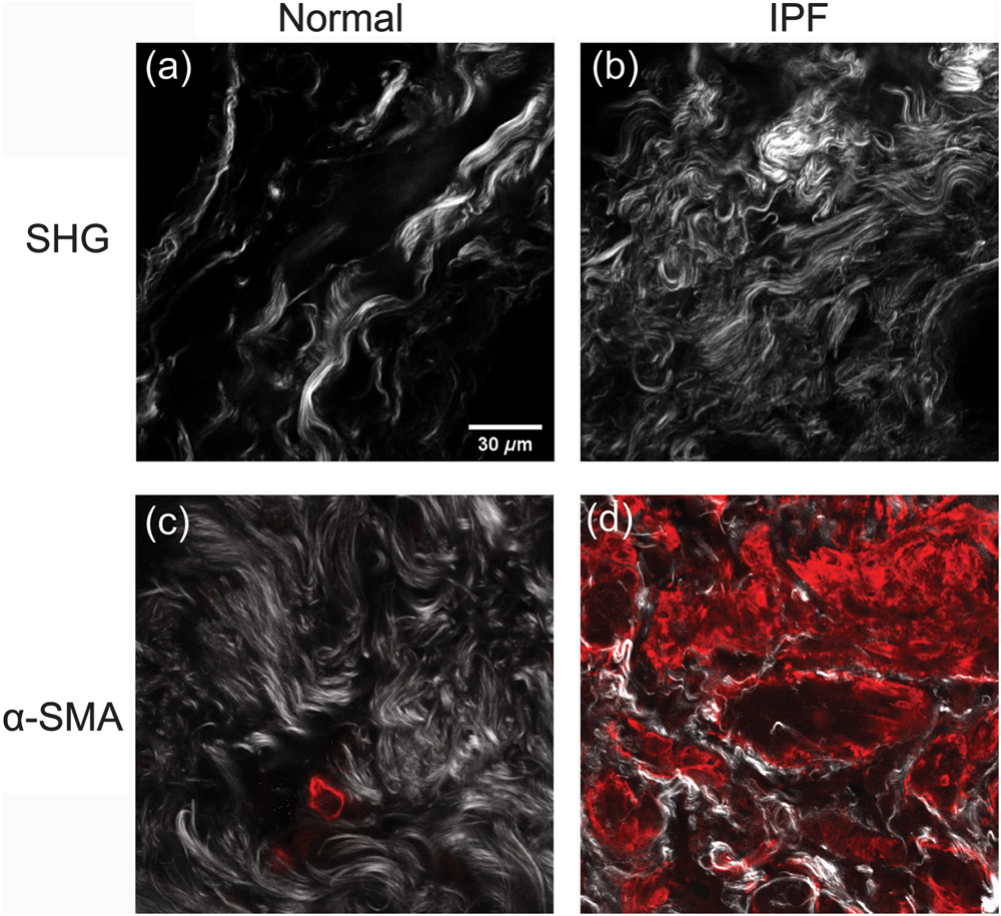
Combined collagen and α-SMA staining in normal [(a) and (c)] and IPF [(b) and (d)] tissues. The top row gives SHG images only and the bottom row is an overlap of SHG (grayscale) and TPEF (red) for α-SMA, identifying fibrotic regions. Scale bar=30  μm.

We first present an overall comparison of the collagen content in normal and IPF lung tissues. The top row of Fig. 1 shows representative SHG images of normal and IPF lung tissues. We note that the IPF tissue has greater coverage across the field and denser collagen accumulation in comparison to normal. IPF also appears to have thinner, wavier fiber structures, whereas the fibers are more linear in normal lung tissue. We point out that collagen morphologies tend to vary significantly across sampling areas, where regions of diseased tissue may resemble that of normal/healthy lung tissue. Still, we previously were able to differentiate these tissues with high accuracy using machine learning analysis of the SHG images.^4^ In order to validate the apparent difference in coverage, we calculated a packing efficiency for each group, where this is quantified by creating a binary mask over a lower threshold of 15 counts (on a 12-bit image stack) and then calculating the fraction of the resulting nonvanishing pixels. We found that IPF has a significantly higher packing (normal: 0.56±0.03, IPF: 0.69±0.04; p<0.01).

We next verified that we were interrogating fibrotic regions, where this is important given the heterogeneity of the collagen morphology in both types of tissues. A standard marker for fibrosis is increased α-SMA expression, as this is associated with a fibroblast-to-myofibroblast transition, where myofibroblasts secrete significantly larger amounts of collagen and other ECM proteins than undifferentiated fibroblasts.^25^^,^^26^ We thus overlapped SHG and TPEF images from an anti α-SMA antibody, where this is shown in the bottom row of Fig. 1. The normal and IPF lungs show little and extensive α-SMA expression, respectively.

**Fig. 2:**
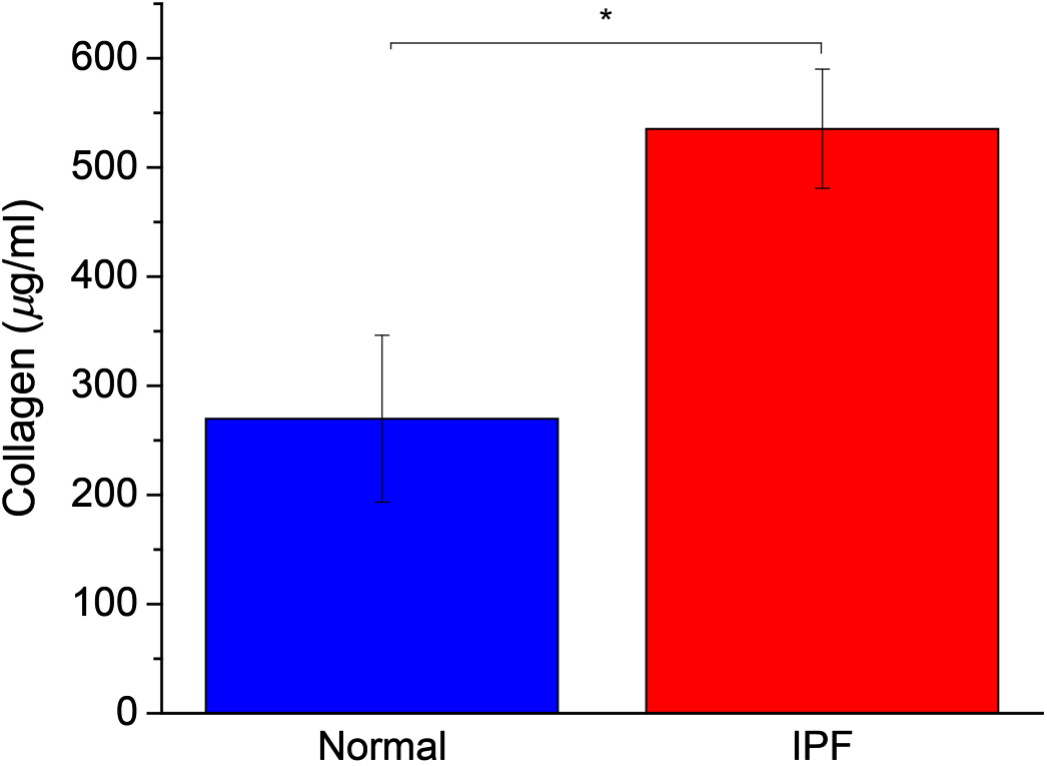
Collagen concentration data of normal (blue) and IPF (red) lung tissues. Standard error bars are shown. Number of samples were six for both IPF and normal with three separate slices in each case. Note: * indicates p<0.05.

We further measured the collagen concentrations for IPF and normal lung tissues using a Sirius Red detection kit. The Sirius Red dye binds to the repeating Gly-X-Y pattern that forms the majority sequence in the fibrillar collagen triple helix. The measured concentrations are shown in Fig. 2, and we found approximately a two-fold increase in collagen concentration in IPF over normal tissues, in agreement with the SHG imaging data showing increased coverage.

### 3.2 Bulk Optical Parameter Measurements

We further characterized the respective normal and IPF tissue structures through the wavelength dependence of the scattering coefficient, $\mu_{s}$, the single scattering anisotropy, g, and the reduced scattering coefficient, $\mu_{s}^{\prime }$. We will also use these values in the Monte Carlo simulations in Sec. 3.3. The scattering coefficient, $\mu_{s}$, is associated with the density and polarizability of a tissue and is equal to the reciprocal of the mean free path of a photon before scattering. We used a set of wavelengths (390, 495, 545, 780, 990, and 1070 nm) that are near those of the SHG (445 nm) and fundamental (890 nm), and as examples, the latter are given in Table 1. We found the IPF tissues had consistently higher values, corresponding to increased densities, consistent with the analyses in Sec. 3.1. The anisotropies were not significantly different between the tissues but increased at longer wavelengths, in good accordance with Mie theory.

**Table 1:** Bulk optical parameters for normal and IPF tissues tabulated as mean±standard error.

Group	λ (nm)	$\mu_{s}$ (${\mathrm{cm}}^{-1}$)	$g_{\text{single}}$	$\mu_{s}^{\prime }$ (${\mathrm{cm}}^{-1}$)
Normal (N=20)	445	327±23	0.914±0.004	26.5±1.6
	890	260±21	0.950±0.003	13.0±1.0
IPF (N=18)	445	405±23	0.908±0.007	41.9±4.2
	890	283±24	0.946±0.006	13.7±1.9

**Fig. 3:**
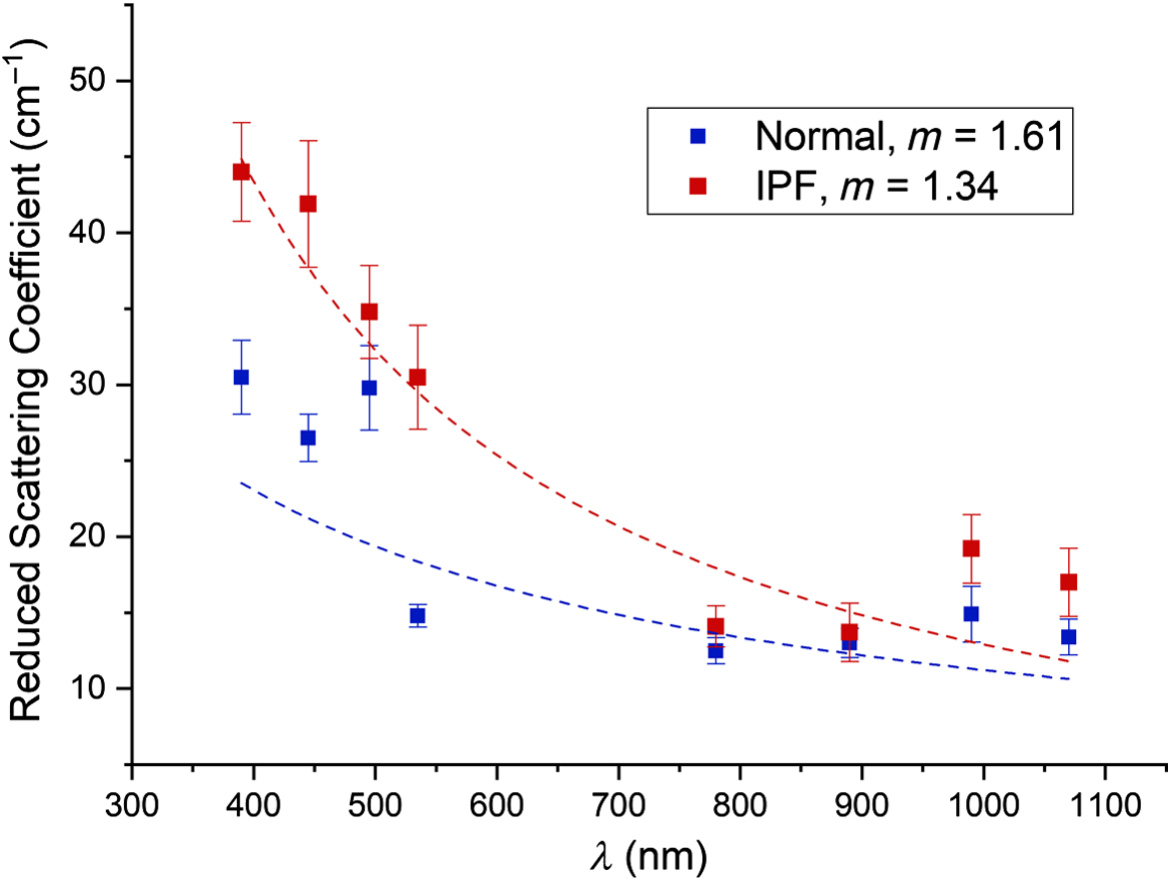
Spectral dependence of $\mu_{s}^{\prime }$ over UV/Vis and NIR wavelengths for normal and IPF tissues where the fit is to the Whittle–Matérn correlation function. The IPF tissues are more highly scattering but have a stronger spectral slope (lower m), indicting a broader range of scatter sizes. There were ∼20 independent measurements at each wavelength using the different tissues.

For quantitation, we compare the spectral dependence of the reduced scattering coefficient, $\mu_{s}^{\prime }$, which arises from the spatial distribution of the refractive index due to structural differences on size scales smaller than the diffraction limit. We follow the treatment of Backman using the Whittle–Matérn correlation function, where the spectral dependence is given by $\mu_{s}^{\prime }(\lambda )∼\lambda^{\left(2m-4\right)}$. Here, the output is the shape factor m, which corresponds to one half of the fractal dimension,^27^^,^^28^ where higher m values are associated with larger, more ordered structures on the approximate size scale of 50 nm to 1  μm. The resulting $\mu_{s}^{\prime }(\lambda )$ values are plotted in Fig. 3, where the best fits for the normal and IPF tissues were m=1.61 and 1.34, respectively. In this analysis, this is a large difference that corresponds to very different tissue structures, specifically indicating IPF tissues have a larger distribution of scatter sizes that contribute to the response. We note that we observed similar behavior in comparing normal and malignant ovarian tissues, where the latter had higher values of $\mu_{s}^{\prime }$ due to increased collagen deposition but stronger wavelength dependence, i.e., lower m due to the reduced regularity of the fibril structure.^29^

### 3.3 Characterization of Fibril Assembly by Local SHG Emission Directionality

SHG in tissues is characterized by nonideal phase-matching, i.e., $\Delta k=k_{2\omega }-2k_{\omega }\neq -0$, where ω and 2ω correspond to the fundamental and SHG angular frequencies. As a consequence, to conserve momentum, this results in a distribution of forward ($F_{\mathrm{SHG}}$) and backward ($B_{\mathrm{SHG}}$) emitted components where we have dubbed the quantity $F_{\mathrm{SHG}}/B_{\mathrm{SHG}}$ as the creation ratio or emission directionality.^21^ In this treatment, lower values correspond to greater phase-mismatch and more disorganized structures relative to the size scale of $\lambda_{\mathrm{SHG}}$. The SHG emission becomes coupled with scattering during tissue propagation and Monte Carlo simulations using $\mu_{s}^{\prime }$ at $\lambda_{\mathrm{SHG}}$ are used to extract the creation ratio as previously described.^20^ As we are interested in the heterogeneity within the tissues, the analysis is performed on 15×15  pixel patches instead of the whole field of view. In previous work, we found this size range to be optimal in examining heterogeneity.^24^

**Fig. 4:**
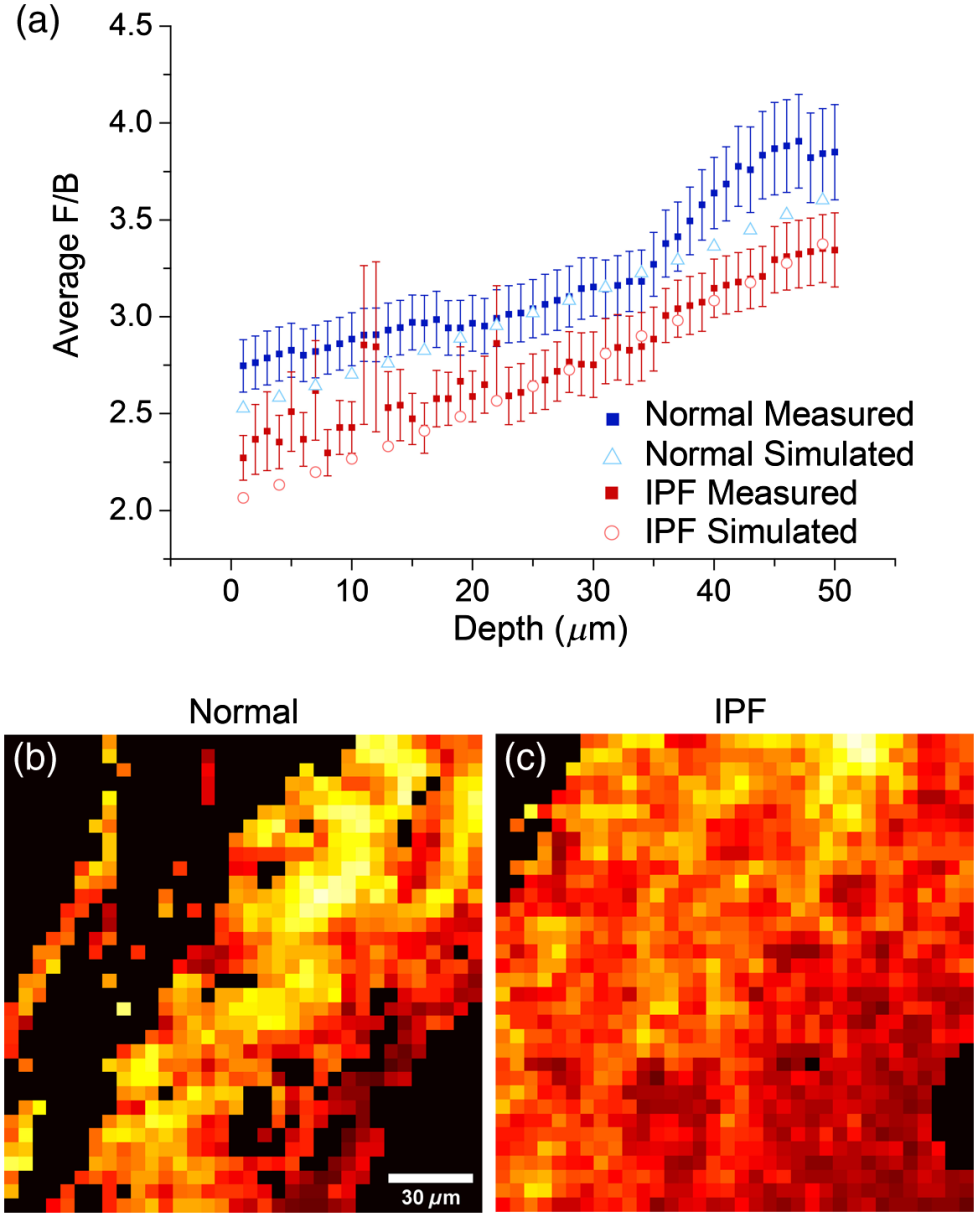
Local analysis of the SHG emission directionality. (a) Measured F/B as a function of depth into normal (blue) and IPF (red) tissues; solid and open symbols correspond to measured and simulated responses, respectively. The resulting $F_{\mathrm{SHG}}/B_{\mathrm{SHG}}$ in 15×15  pixel patches for (b) normal and (c) IPF tissues. Number of stacks were 34 and 75 for IPF and normal, respectively. Scalebar=30  μm.

The measured F/B versus depth and best simulation for the creation ratio, $F_{\mathrm{SHG}}/B_{\mathrm{SHG}}$, are shown in Fig. 4(a) and summarized in Table 2. We note that at a few depths, there are similar experimental F/B values between the normal and IPF tissues. This is likely due to nonideal F/B behavior for some of the IPF samples due to intrinsic heterogeneity of these tissues relative to the normal lung. Still, the extracted best-fit values of the SHG creation ratio, $F_{\mathrm{SHG}}/B_{\mathrm{SHG}}$ and associated reduced chi-squared values are negligibly affected. The locally extracted $F_{\mathrm{SHG}}/B_{\mathrm{SHG}}$ values for the patches of representative images for normal and IPF are shown in Figs. 4(b) and 4(c), respectively. Compared to normal, IPF has a lower $F_{\mathrm{SHG}}/B_{\mathrm{SHG}}$ creation ratio, which suggests smaller and less organized collagen fibrils in the axial direction relative to $\lambda_{\mathrm{SHG}}$.

**Table 2:** Creation ratios for normal and IPF lung, tabulated as mean±SE.

Group	$F_{\mathrm{SHG}}/B_{\mathrm{SHG}}$	$F_{\mathrm{SHG}}/B_{\mathrm{SHG}}$ std. dev.^a^
Normal (N=75)	2.67±0.14	1.16±0.07
IPF (N=34)	2.02±0.12	0.74±0.07
p-Value	<0.01	<0.01

a Calculated per individual tissue.

We propose that the lower organization of collagen in IPF is either due to fibrosis (i.e., fibrotic collagen is intrinsically less organized) or other changes in the ECM that affect the collagen morphology. This finding is in agreement with previous SEM studies that showed that the collagen fibrils were more irregular in IPF than in normal tissue.^29^ We also examined the heterogeneity within these tissues. Interestingly, we found a lower $F_{\mathrm{SHG}}/B_{\mathrm{SHG}}$ standard deviation for IPF, which suggests that Δk values are more uniform within the obtained stacks. Thus, while overall the IPF tissues have more heterogeneity, containing both normal and fibrotic regions as well, the fibrotic regions themselves are more uniform than normal tissues.

### 3.4 Polarization-Resolved Second Harmonic Generation

There are previous accounts using immunostaining that showed the relative proportion of Col I and Col III is different in IPF relative to normal lung. Specifically, Col III is relatively increased in early stage disease, and then Col I is more prevalent in later stages, corresponding to an older scar.^25^^,^^30^^,^^31^ Immunostaining is not always very quantitative in general and less so for the current case as Col III antibodies have crosstalk with those for Col I as the epitope is the same.

To examine this proposed change in relative collagen isoform abundance, we employed SHG polarization analysis based on the single-axis molecular model, which is sensitive to the α-helical pitch angle.^11^ Based on structural biology, the pitch angle (angle of coil relative to long molecular axis) for Col III is about 2 deg higher than that of Col I. Previously, we showed that SHG could discriminate between the fibrillar morphology of varying collagen I/collagen III concentrations in mixed gels.^10^ We also successfully differentiated these gels based on the extracted pitch angles, where the results were consistent with known difference of Col I and III from the protein database.^32^

**Fig. 5:**
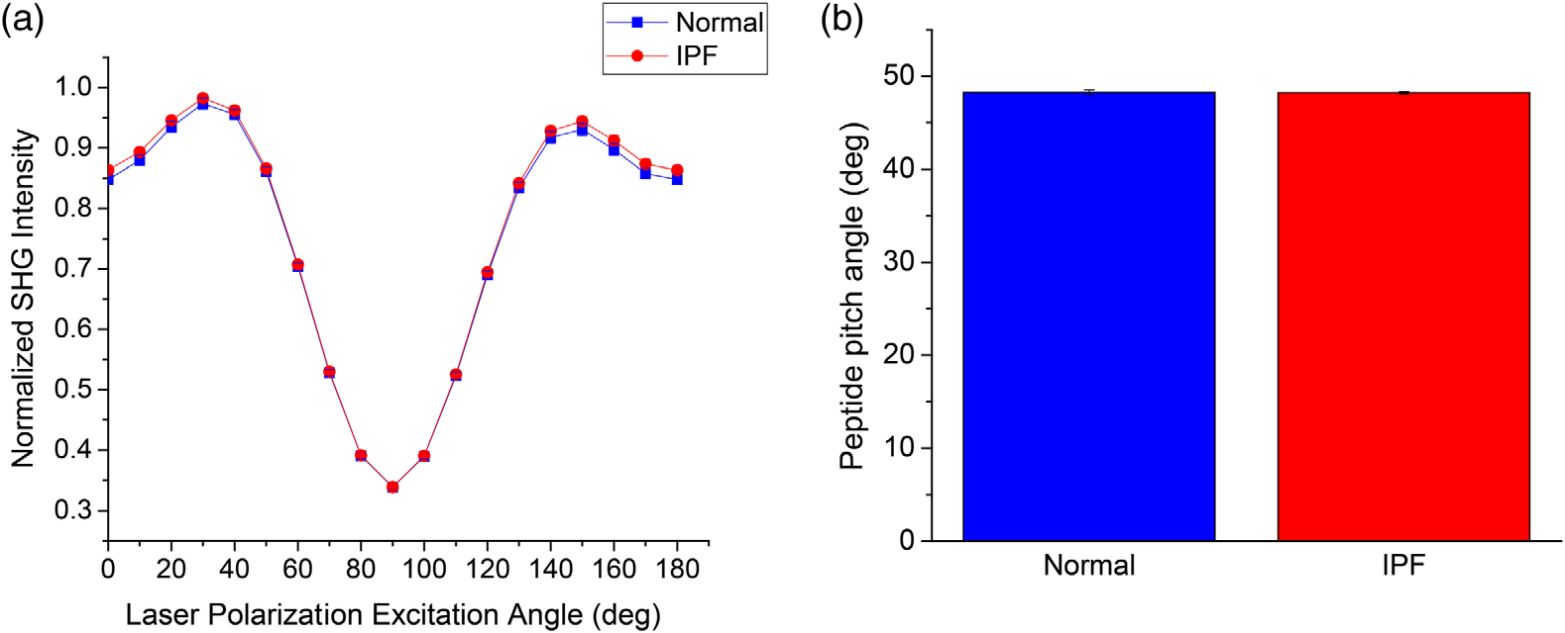
Linear polarization analysis of normal (blue) and IPF (red) tissues. (a) The reconstructed pixel-based response; (b) the extracted pitch angles. The data were similar to each other, inconsistent with an increase in Col III abundance in IPF.

Applying this same polarization-resolved SHG technique to image human lung tissues [Eq. (1)], we obtained pitch angles (Fig. 5) of 48.25 and 48.2 for normal and IPF, respectively, suggesting there is no measurable collagen isoform change in IPF. This could occur as there is no significant increase in Col III or it is not identifiable due to the spatial heterogeneity in IPF.

**Fig. 6:**
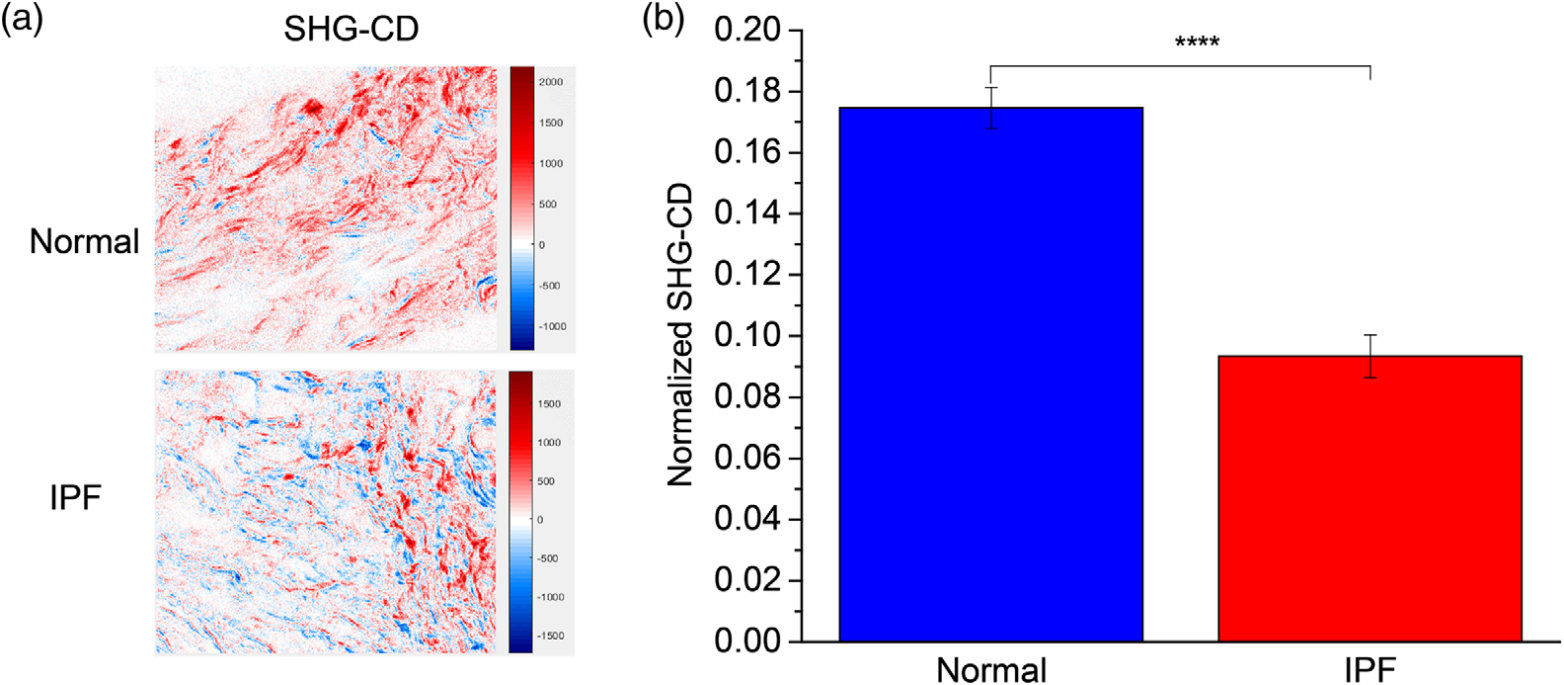
(a), (b) Normalized SHG-CD data of cleared normal (blue) and IPF (red) lung tissues measured at 780-nm excitation wavelength. (a) The red and blue correspond to positive and negative SHG-CD values, respectively, which are determined by the fiber polarity. (b) Standard error bars are shown. Number of unique images were 134 and 121 for IPF and normal, respectively. Field size=85×85  μm. Note: **** represents p<0.00001.

We further analyzed the helical properties of normal and IPF tissues via the SHG-CD protocol as described previously [Eq. (2)].^5^ Representative SHG-CD images are shown in Fig. 6(a) for both normal and IPF, where red and blue correspond to the sign of the response and corresponds to the polarity of the fiber and the magnitude arises from the alignment of the triple helices within the focal volume.^5^ The response is calculated using the absolute value [Eq. (2)]; we found the average SHG-CD was significantly higher (almost two-fold) in normal versus IPF [Fig. 6(b)]. This decreased chirality in IPF suggests either improper collagen fibril formation or changes in crosslinking. In principle, this would also be consistent with an increase in Col III; however, that is not consistent with the pitch angle analysis (Fig. 5).

## 4 Discussion

While seemingly paradoxical, the collagen macro/supramolecular changes in IPF are not well studied. SHG can directly visualize the collagen assembly, is sensitive to morphological changes within the assembly, and has significant potential, especially when coupled with polarization-resolved approaches and further with optical scattering measurements.^33^ For example, the SHG directional analysis of the measured F/B versus depth response combined with Monte Carlo simulations based on optical properties yields data on the relative fibril size and assembly that is consistent with the (limited) available SEM measurements.^29^ Moreover, the spectral analysis of the reduced scattering coefficient revealed that IPF is both more dense (higher $\mu_{s}$, packing efficiency) and less organized (lower m) than normal lung tissue, further consistent with the lower SHG creation ratio, $F_{\mathrm{SHG}}/B_{\mathrm{SHG}}$. Thus, these optical measurements are consistent with both increased collagen deposition and decreased order than that of normal tissues. Importantly, both the SHG and optical properties can be performed on intact tissues, where thin sections are required for any high-resolution electron microscopy work.

We did not find any differences in the respective α-helix pitch angle and thus no difference in relative Col I/III abundance. It is possible that due to the heterogeneity within the IPF tissues, our imaging regions were not optimized. However, the SHG-CD response was very different, so there were clear changes in chirality in these locations. Moreover, there was clear enhanced α-SMA expression in these tissues (Fig. 1), consistent with fibrotic regions. It is now known there is marked difference in crosslinking in IPF relative to normal tissues,^34^ where this could affect the net chirality. There are other possible underlying ECM changes as well, e.g., increased fibronectin deposition,^35^ to which SHG is not directly sensitive.

Interestingly, we found a similar trend in our work on ovarian cancer, where Col III was also reported to be increased.^36^ Similarly, this was not borne out by extracted pitch angles while the SHG-CD was significantly less for ovarian cancer than normal stroma.^6^ Moreover, the SHG directional analysis and optical properties analysis all trended in the same direction (i.e., denser and more disordered) for IPF and high grade ovarian cancer relative to the corresponding normal tissues, suggesting commonalities in the misformed collagen. Intriguingly, several of the same pathways are altered in both diseases, e.g., upregulation of proteases.^36^^–^^38^ Moreover, the microenvironments of fibroses and cancers have many similarities including fibroblast activation, increased collagen synthesis, and stiffness.^37^ SHG is thus sensitive to a range of physical changes associated with increased collagen deposition accompanying different diseases.

## 5 Conclusions

IPF prognosis is poor due to the lack of effective treatment options, limited knowledge of the disease etiology and underlying molecular and temporal changes associated with disease progression.^4^ To help solve this problem, we have used SHG imaging in combination with optical property measurements to examine macro/supramolecular and fibril changes in the fibrotic collagen. These metrics indicated significant differences in collagen assembly between the normal and IPF tissues, with the latter being characterized by increased disorder, where this is consistent with the limited available structural biology data.^29^ As SHG can be done on whole tissues, the ability to obtain subresolution structural data without the constraints of historical methods offers great promise for this imaging modality as a diagnostic tool. For example, a laser scanning microendoscope^39^ could be developed to monitor disease progression as well as response to treatment.
